# Changes in body composition in early breast cancer patients treated with aromatase inhibitors

**DOI:** 10.1007/s40618-024-02401-7

**Published:** 2024-06-10

**Authors:** R. Pedersini, G. Schivardi, L. Laini, M. Zamparini, A. Bonalumi, P. di Mauro, S. Bosio, V. Amoroso, N. Villa, A. Alberti, N. Di Meo, C. Gonano, B. Zanini, M. Laganà, G. Ippolito, L. Rinaudo, D. Farina, M. Castellano, C. Cappelli, E. L. Simoncini, D. Cosentini, A. Berruti

**Affiliations:** 1 Medical Oncology Department, ASST Spedali Civili of Brescia, Piazzale Spedali Civili 1, 20123 Brescia, Italy; 2 SSVD Breast Unit, ASST Spedali Civili of Brescia, Brescia, Italy; 3 Department of Medical and Surgical Specialties, Radiological Sciences and Public Health, Medical Oncology, University of Brescia, ASST Spedali Civili, Brescia, Italy; 4 Department of Clinical and Experimental Sciences, University of Brescia, Brescia, Italy; 5 Tecnologie Avanzate Srl, Turin, Italy; 6 Department of Internal Medicine and Endocrinology, University of Brescia, ASST Spedali Civili, Brescia, Italy

**Keywords:** Breast cancer, Body composition, Fat body mass, Lean body mass, Aromatase inhibitors

## Abstract

**Purpose:**

The aim of the study was to analyze the modification of total and regional body composition in early breast cancer patients treated with aromatase inhibitors (AIs).

**Methods:**

This is a prospective, single-center, observational, longitudinal study. Four-hundred and twenty-eight patients treated with adjuvant aromatase inhibitors were enrolled at the Medical Oncology and Breast Unit of Spedali Civili Hospital in Brescia from September 2014 to June 2022. Several body composition parameters including total and regional fat and lean body mass were investigated with dual-energy X-ray absorptiometry (DXA) scan at baseline and after 18 months of treatment with aromatase inhibitors.

**Results:**

A significant increase in fat body mass (mean + 7.2%, 95% confidence interval [CI]: 5.5;8.9%) and a reduction in lean body mass (mean −3.1%, 95% CI −3.9; −2.4) were documented in this population. The changes in fat and lean body mass varied considerably according to different body districts ranging between + 3.2% to + 10.9% and from−1.3% to −3.9%, respectively.

**Conclusion:**

Aromatase inhibitor adjuvant therapy in early breast cancer is associated with changes in body composition, with a wide variability among different body districts, leading to a risk of sarcopenic obesity. Supervised physical exercise that focuses on single body parts that may display detrimental variations may be beneficial for AIs treated patients.

**Supplementary Information:**

The online version contains supplementary material available at 10.1007/s40618-024-02401-7.

## Introduction

Breast cancer is the most common cancer in women and is currently the leading cause of cancer-related mortality in females worldwide [1]. The improvement of the screening programs and the advances in adjuvant therapies have led to a decline in breast cancer mortality [2]. Since the 5-year survival rate of BC patients is around 90% [3], it is important to focus on their overall well-being and quality of life [4]. It has been estimated that more than 50% of early breast cancer (EBC) survivors has a significant weight gain after diagnosis [5], and strategies in preventing the obesity risk are included in the American Cancer Society breast cancer survivorship care guidelines [4]. Adjuvant chemotherapy is widely recognized to be an independent predictive factor for weight gain in EBC patients [5–7]. Few studies have investigated the impact of endocrine therapies, such as tamoxifen or aromatase inhibitors, on body weight or body mass index (BMI) with mixed results [8–11].

BMI is a simple yet valid tool for assessing overall adiposity at population levels; however, it is subject to obvious limitations because it fails to discriminate between two distinct compartments of body composition, such as lean body mass and fat mass [12–15]. Body composition measurement may offer additional information to quantify the risk of several diseases beyond that of body weight or BMI alone [16]. Excess fat mass, in fact, increases the risk of cancer recurrence, type 2 diabetes, and cardiovascular diseases [17, 18] whereas low lean mass increases the risk of frailty, falls, and functional decline [19, 20]. In addition, changes in body composition were found to be associated with an increased risk of death in overall population [18] and breast cancer survivors [20–22]. Aromatase inhibitor (AI)-therapy, through inhibition of aromatase enzyme, induces a deep reduction in circulating estrogen levels associated with a potential increase of androgen and progesterone levels [23]. This hormonal derangement could have a significant impact on body composition, regardless of weight gain and BMI. However, evidence coming from small or retrospective studies provided controversial results [23, 24].

The aim of this prospective longitudinal study is to assess the modification of whole and regional body composition parameters in a large series of EBC patients after 18 months of AI-therapy.

## Patients and methods

This is a prospective, single-center, longitudinal, observational study conducted at the Medical Oncology Unit and Breast Unit of Azienda Socio Sanitaria Territoriale (ASST) Spedali Civili of Brescia (Italy) from September 2014 to June 2022, approved by the local Ethic Committee (internal protocol n° 3270, registered in August 2014). This study followed the Strengthening the Reporting of Observational Studies in Epidemiology (STROBE) reporting guideline [25].

The main objective of this research was to assess the occurrence of incident morphometric vertebral fractures in EBC patients who were treated with AIs. We are presenting the outcomes of the secondary objectives of the study, which were to evaluate the changes in total FBM and LBM in the same population. Body composition of the different body districts such as: head, trunk, left and right arm, left and right leg and of other parameters of body compositions were explored.

Key eligibility criteria were the following: 1) histologically confirmed EBC; 2) eligibility to adjuvant treatment with AIs; 3) willingness to adhere to the study protocol by signing the consent form. Previous chemotherapy was allowed, but previous tamoxifen was not. Radiotherapy after surgery was allowed, if indicated. Patients with poor performance status (PS ECOG > 2), comorbidities, poor compliance (i.e. patients not motivated to follow the scheduled activities mandatory for the study or patients living far away from our hospital), metastatic disease or previously treated for other tumors were excluded.

DXA measurements [26–28] were performed at baseline and after 18 months, using Hologic Delphi instrumentation (Hologic Corporation, Waltham, Massachusetts) and APEX Software version 4.6. Data were analysed by dedicated endocrinologists (AD and CC) and radiologists (ND and DF). They were blinded to patients’ clinical information and DXA scan’s time point.

FBM and LBM were assessed for whole body and several districts at two time-points: baseline, before starting AI-therapy, and after 18 months of AI-therapy.

DXA measures of adiposity [29] and lean mass included: Fat Mass index (FMI: total FM/height2); Visceral Adipose Tissue (VAT), VAT mass, VAT volume, VAT area, android-fat %, gynoid-fat %, android/gynoid fat ratio, Lean Mass index (LMI, total LM/height2); Appendicular Lean Mass index (ALMI, lean mass from arms plus legs/height2). These parameters were also collected at baseline and after 18 months of AI-therapy.

Height was measured with a stadiometer to the nearest 0.1 cm. Weight was measured to the nearest 0.1 kg with a calibrated scale while wearing light clothes. BMI was calculated as weight kg/height[m]^2^. Patients were categorized for smoking habits as previous, current, or never smokers; alcohol consumption as less or more than 12 g per day; weekly physical activity as mild, moderate, or intense physical exercise.

### Statistical method

Categorical variables were expressed as frequencies and percentages; the distribution of continuous variables was calculated as mean ± 95 confidence interval (95% CI) or as median and range.

The difference of body composition over time (from baseline to the last control after 18 months of AI-therapy) were tested by paired t-test (or Wilcoxon signed-rank test when necessary): changes were expressed as percentage differences between 18-months and baseline and 95% Confidence Interval (CI). To investigate possible determinants and predictors of changes in body compositions we used univariable and multivariable linear regression models. Variables that have reached a p level of significance < 0.10 at univariable analysis were included in the multivariable model.

All the analyses were proformed with SPSS (IBM Corp. Released 2015. IBM SPSS Statistics for Windows, Version 23.0. Armonk, NY: IBM Corp.), considering a maximum I type error of 5% (two-tailed).

### Power calculation

In our paper, we utilized the “pwr” package in R to conduct power calculations, considering an 80% power and a Type I error rate of 5%. Our objective was to determine the minimum effect size needed to reject the null hypothesis of no effect, utilizing data from 347 patients.

These power calculations were performed using a paired t-test and assuming a two-sided alternative hypothesis. The results provide valuable insights into minimum detectable significant effect size, that with our sample size, considering the specified power and significance levels, correspond to 0.05.

## Results

### Patients’ characteristics

From September 2014 to June 30, 2022, 460 consecutive Caucasian women with hormone receptor (HR)-positive EBC, followed at the Medical Oncology and Breast Unit of the ASST-Spedali Civili of Brescia were assessed for eligibility. As depicted in the consort diagram reported in supplementary information (Online Resource 1), 15 patients were excluded from the study for refusal or ineligibility and 5 patients for other reasons. Four hundred and forty patients, meeting the eligibility criteria, entered the study; 12 patients however were excluded: 6 patients did not perform the second DXA scan on scheduled time point and other 6 patients were lost at follow-up after the first DXA scan.

Therefore 428 patients were fully evaluable for DXA-derived bone parameters and 347 patients were evaluable for body composition parameters such as FBM, LBM, ALMI, trunk appendicular fat ratio, lean/ht^2^, and FMI. In an explorative analysis involving 216 patients’ android fat, gynoid fat, android/gynoid fat ratio were evaluated and in 66 patients also VAT was assessed.

The characteristics of the 428 patients were reported in Online Research 2 of the supplementary information and that of 347 patients is reported in the Table 1.

**Table 1:** Characteristics of the enrolled patients (N = 347)

Characteristics of the 347 patients	N° (%)
Median age (range)	60.5 (28–84)
Menopausal status	
Pre-	72 (16.8%)
Post-	356 (83.2%)
Physical activity	
Yes	75 (21.6%)
No	272 (78.4%)
Smoke	
Yes	80 (23.1%)
No	367 (76.9%)
Alcohol consumption	
Yes	75 (21.6%)
No	272 (78.4%)
pT	
1	232 (66.9%)
≥ 2	115 (44.1%)
pN	
0	213 (61.4%)
≥ 1	134 (38.6%)
Histological type	
No Special Type (NST)	245 (70.6%)
Other	102 (29.4%)
Grading	
G1 o G2	182 (52.4%)
G3	165 (47.6%)
HER 2 status	
Positive	59 (17.0%)
Negative	288 (83.0%)
Chemotherapy	
Yes	145 (41.8%)
No	202 (58.2%)
Radiotherapy	
Yes	230 (66.0%)
No	117 (34.0%)

Characteristics of the 347 patients	Mean (95% CI)
ER (%), mean (95% CI)	95.6 (94.4–94.7)
PgR (%), mean (95% CI)	61.4 (57.5–65.4)
Ki67 (%), mean (95% CI)	23.3 (21.7–24.7)

*N*° number of patients, % percentage of patients, *CI* confidence interval, *pT* pathological tumor stage, *pN* pathological nodal status

Median age was 60.5 years (range 28–84). The proportion of pre- and post-menopausal women respectively was 16.8% and 83.2%. Regarding lifestyle, 21.6% of patients were physically active, 23.1% were smokers, and 21.6% consumed alcohol. Nearly 66.9% of the patients was staged as pT1, 61.4% as N0 (N = 213); 47.6% presented a G3-tumour. Mean Ki67 was 23.3% and 17% of the patients had a HER2-positive tumor. Median rate expression of Estrogen Receptor (ER) and Progesterone Receptor (PgR) were 95.6% and 61.4%, respectively. Nearly 41% of the patients had received adjuvant chemotherapy and 66% complementary radiotherapy.

### Changes in weight, BMI, fat body mass and lean body mass for whole body and regional districts during adjuvant AI-treatment

Weight, BMI, fat and lean body mass of whole body and regional districts at baseline and after 18 months from starting AI-therapy are reported in Table 2. At baseline, mean weight was 66 kg (95% CI 64.7;67.3) and mean BMI was 25.2 (95% CI 24.8;25.8). Weight slightly increased after 18 months of AI-therapy of about 0.6% from 66 kg (95% CI 64.7;67.3), to 66.3 kg (95% CI 65;67.7), as well as BMI that increased from 25.2 (95% CI 24.8;25.8) to 25.4 (95% CI 24.9;25.9). LBM of the whole body decreased significantly from 39,916 g (95%CI 39377;40,456) to 38,609 g (95% CI 38058;39,160, p < 0.001 [mean decrease −3.1%]), while total FBM increased from 24,647 g (95% CI 23751;25,544) to 25,961 g (95% CI 25061; 26,861, p < 0.001 [mean increase + 7.2%]).

**Table 2:** Changes of weight, BMI, total and regional lean and fat body mass before and after adjuvant AI treatment

Anthropometric parameters	Number of patients	Baseline Mean (95% CI)	After 18 months Mean (95% CI)	Change from baseline % (95% CI)	*p*
Weight (Kg)	347	66.0 (64.7;67.3)	66.3 (65;67.7)	.6 (.1;1.3)	.036
BMI (Kg/h2)	347	25.2 (24.8;25.8)	25.4 (24.9;25.9)	.6 (.1;1.3)	.036

Lean mass gr		Baseline Mean (95% CI)	After 18 months Mean (95% CI)	Change from baseline mean % (95% CI)	*P*
Lean mass	347	39,916 (39,377;40,456)	38,609 (38,058;39,160)	−3.1 (−3.9; −2.4)	< .001
Trunk lean mass	347	20,736 (20,427;21,045)	19,892 (19,560;20,224)	−3.8 (−4.9; −2.8)	< .001
Left arm lean mass	347	1876 (1843;1908)	1819 (1785;1853)	−2.5 (−3.8; −1.3)	< .001
Right arm lean mass	347	1992 (1958;2026)	1956 (1920;1991)	−1.3 (−2.6; −.1)	.005
Left leg lean mass	347	6100 (5993;6207)	5936 (5828;6043)	−2.4 (−3.4; −1.5)	< .001
Right leg lean mass	347	6198 (6087;6310)	6036 (5927;6145)	−2.2 (−3.3; −1.0)	< .001
Head lean mass	347	3022 (2987;3058)	2887 (2850;2925)	−3.9 (−5.2; −2.5)	< .001

Fat mass gr Mean (95% CI)		Baseline Mean (95% CI)	After 18 months Mean (95% CI)	Change from baseline mean % (95% CI)	P
Fat mass	347	24,647.3 (23,751;25,544)	25,961.0 (25,061;26,861)	7.2 (5.5;8.9)	< .001
Fat mass (%)	347	36.2 (35.4;36.9)	38.2 (37.5;38.9)	6.8 (5.5;8.1)	< .001
Trunk fat mass	347	11,825 (11,272;12,379)	2632 (12,087;13,177)	10.9 (8.4;13.5)	< .001
Left arm fat mass	347	1562 (1470;1653)	1593 (1525;1661)	7.2 (4.6;9.8)	< .001
Right arm fat mass	347	1552 (1484;1620)	1656 (1496;1816)	8.0 (2.3;13.7)	.011
Left leg fat mass	347	4272 (4116;4429)	4470 (4307;4634)	6.0 (4.2;7.8)	< .001
Right leg fat mass	347	4471 (4307;4635)	4633 (4462;4804)	8.7 (.2;17.2)	< .001
Head fat mass	347	891 (879;903)	911 (898;924)	3.2 (1.5;4.8)	.001

*AI* aromatase inhibitors, *BMI* body mass index, *CI* Confidence interval, *p* p-value

Univariable and multivariable linear regression analysis of the association between risk factors and percentage change in FBM and LBM (considered as continuous) are presented in Supplementary tables. Univariable analysis resulted in a direct association between FBM (g) and age (B −0.5, 95% CI −0.6;−0.3, p < 0.001), premenopausal status (B 15.2, 95% CI 10.8;19.5, p < 0.001) and chemotherapy (B 5.3, 95% CI 1.9;8.8, p < 0.001). Only age (B −0.2, 95% CI −0.4;−0.1, p = 0.03) and premenopausal status (B 10.4, 95% CI 4.8;16.0, p =  < 0.001) were independent variables at multivariable analysis (Online Research 3). Premenopausal status was associated with an inverse decrease in LBM just failing to attain the statistical significance both at univariable (B −1.6, 95% CI −3.6;0.4, p = 0.10) and multivariable analysis (B −1.16, 95% CI −3.6;0.4, p = 0.11). Similarly, a direct relationship (close to the statistical significance) was shown between physical activity and increase in LBM either at the univariable (B 1.6, 95% CI −0.2;3.3, p = 0.09) or the multivariable analysis (B 1.5, 95% CI −0.2;3.3, p = 0.09) (Online Research 4).

**Fig. 1:**
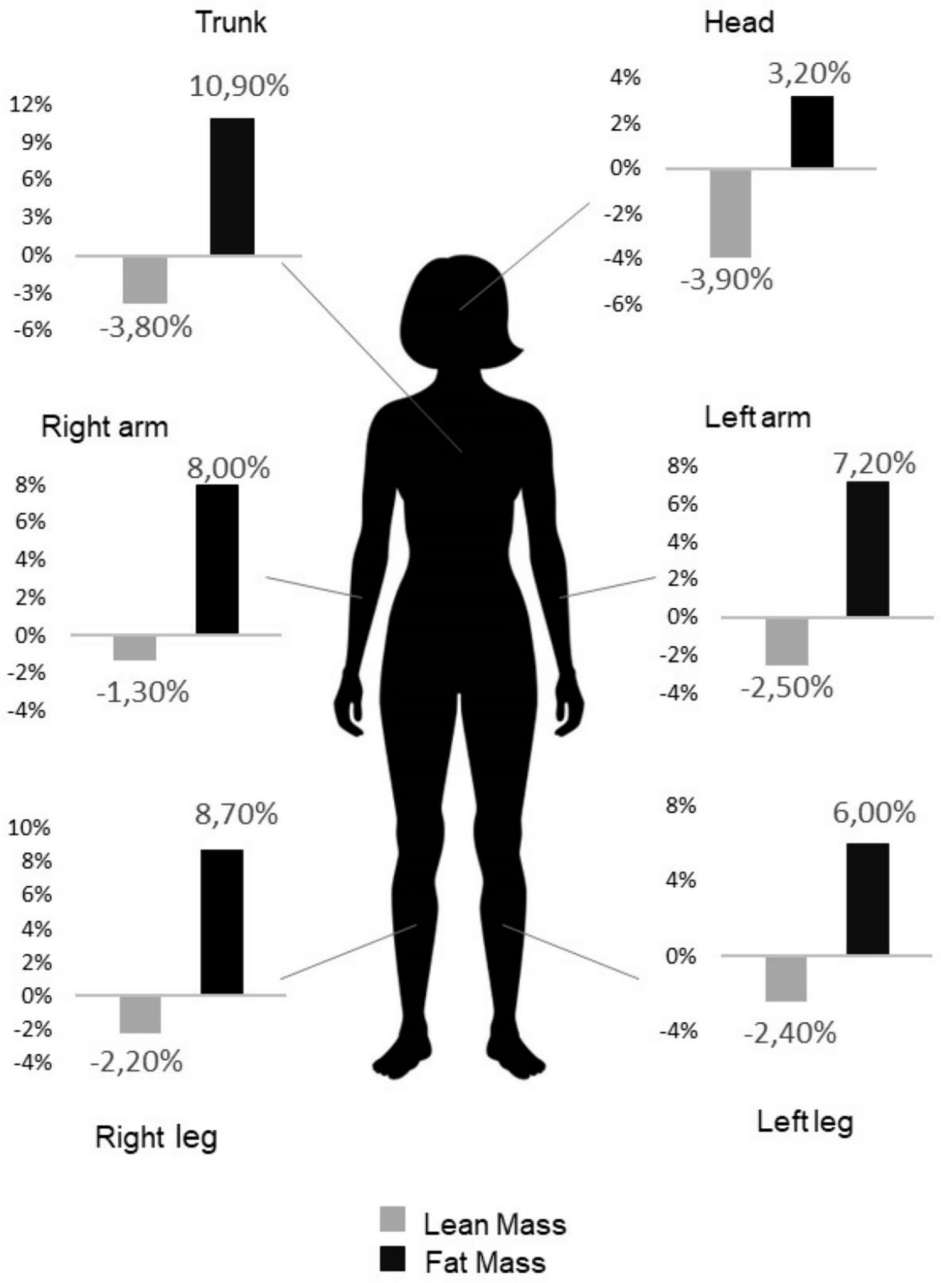
Percent changes in fat and lean mass in different body districts after 18-month of AI-treatment

The changes in body composition in the different body districts varied considerably: LBM decrease varied from −3.9% in the head to −1.3% in the right arm and the increase in FBM varied in a range from + 3.2% in the head to + 10.9% in the trunk (Fig. 1). The lean mass in the left arm declined more noticeably than in the right arm (−2.5% vs −1.3%), but there was no change in lean mass in the two lower limbs. All four limbs experienced a similar increase in fat mass.

### Changes in other body composition parameters during adjuvant AI-treatment

The changes of other anthropometric parameters, assessed at baseline and after 18 months from starting AI-therapy, are reported in Table 3. AIs administration resulted in a 5.6% increase in gynoid fat and an 8.2% increase in android fat, leading to a 2.2% increase in the android/gynoid fat ratio. Also, the trunk/appendicular fat ratio increased considerably by 5.7% and FMI increased by 6.8%. On the other hand, lean mass parameters like lean/h2 and ALMI decreased significantly after AIs by 3.3% and 1.6%, respectively. In the small subgroup where it was evaluated (Table 4), visceral adiposity showed the most significant change. The increase in VAT mass, VAT volume and VAT area in fact increased by 18.9%, 19.4% and 22.1%, respectively.

**Table 3:** Changes of other body composition parameters during AI-Therapy

Parameters Mean (95% CI)	Number of patients	Baseline Mean (95% CI)	After 18 months Mean (95% CI)	Change from baseline mean% (95% CI)	*P*
FMI	347	9.4 (9.0;9.7)	9.8 (9.5;10.1)	6.8 (51;8.6)	0.002
trunk/appendicular fat ratio	347	.98 (.96;1.01)	1.02 (.99;1.05)	5.7 (3.9;7.4)	< .001
Lean/h^2^	347	15.2 (15.0;15.4)	14.7 (14.5; 14.9)	−3.3 (−4.0; −2.5)	< .001
ALMI	347	6.1 (6.0;6.2)	6.0 (5.9;6.2)	−1.6 (−3.2; .0)	< .001
Android-fat%	216	35.6 (34.2; 36.9)	37.8 (36.4; 39.1)	8.2 (5.9; 10.6)	< .001
Gynoid-fat%	216	39.4 (38.6; 40.2)	41.4 (40.6; 42.1)	5.6 (4.2; **7.)**	< .001
android/gynoid fat ratio	216	.89 (.87; .92)	.91 (.88; 93)	2.2 (.8; 3.6)	.0013

*AI* aromatase inhibitors, *FMI* total fat mass to the height, *Lean/h*^2^ total lean mass to the height, *ALMI* appendicular lean mass index, *CI* Confidence interval, *p*: p-value

**Table 4:** Changes in Visceral Adiposity before and after AI therapy in a subset of 66 patients

Parameters Mean (95% CI)	Number of patients	Basaline Mean (95% CI)	After 18 months Mean (95% CI)	Change from baseline % (95% CI)	*p*
VAT mass	66	534.2 (460.9; 607.5)	606.4 (528.0; 684.8)	18.9 (11.4;26.5)	< .001
VAT volume^3^	66	577.5 (498.2; 656.7)	657.2 (572.9; 741.5)	19.4 (11.9;26.9)	< .001
VAT area^2^	66	109.1 (93.8; 124.4)	125.1 (109.1; 141.2)	22.1 (14.1;30.1)	< .001

*AI* aromatase inhibitors, *VAT* visceral adipose tissue, *CI* Confidence interval, *p* p-value

## Discussion

In this relatively large, single-center prospective study, patients with EBC who underwent adjuvant AI-therapy experienced a 7.0% increase in FBM and a 3.0% decrease in LBM after 18 months. These data contrasted with the minimal alterations in body weight and BMI, which revealed a slight increase of 0.6%. Our study underscores the significant limitations of body weight and BMI in the assessment of body composition. DXA has gained interest for estimating FBM and LBM [26, 28, 30]. The assessment of body composition in breast cancer patients undergoing AIs has received limited attention in the literature, and published results have been contradictory. In the paper of Van Soom et al. [31], a significant loss of fat free mass and gain in fat mass percentage (+ 6.4%), without a concomitant altered body weight (+ 1.6 kg), has been observed after 12 weeks of chemotherapy. A retrospective analysis involving sixty-four consecutive patients receiving AI-therapy revealed a mean increase of 9.1% in total adipose tissue area, as assessed by CT scan, without any data on LBM changes [32]. Ali et al. [33] reported significantly greater levels of body fat in women taking tamoxifen and Nguyen et al. [34] al found that fatty liver and intra-abdominal fat were more common among tamoxifen users. Adjuvant treatment with exemestane after at least 2 years of tamoxifen therapy was found to result in a significant decrease in FBM in the study by Francini et al. [24]. According to Van Londen, et al. [35], AIs users showed a maintenance of total body fat, an increase in LBM, and an increase in free testosterone levels.

Our study, which was conducted on a larger case series than previous ones, underscores the risk of sarcopenic obesity faced by women who undergo adjuvant therapy with AIs.

This condition is notoriously associated with a general increase in patient fragility, leading to increased risks of mobility impairment, disability, lower quality of life, and all-cause mortality Sarcopenic obesity also places the patient at greater risk of skeletal fragility [36, 37]. In a previous cross-sectional study led by our group on EBC patients treated with AIs the frequency of vertebral fractures was most associated with the phenotype of low lean mass and high fat mass [37, 38]. In a subsequent study FBM was found to be an independent risk factor of vertebra fracture progression after AIs. Moreover, a recent study conducted on patients with prostate cancer who were undergoing androgen deprivation therapy found a negative correlation between sarcopenic obesity and bone health [39].

The changes in body composition observed in this study are not significantly different from those previously observed in patients with prostate cancer receiving androgen deprivation therapy (ADT) [40, 41]. It is tempting to speculate that women who receive AI-treatment are equally prone to sarcopenia as men who receive ADT. A longer follow-up is required to determine the percentage of patients who may experience sarcopenic obesity after receiving AIs. Anyway, oncologists who are treating EBC patients with AIs should take into account the results of this study. Moreover, in the present study premenopausal status had an independent predictive role of LBM loss after AIs and physical activity was found to be protective. These findings emphasize the significance of physical activity in preventing sarcopenia caused by AIs. It should be advocated for all patients, but particularly for younger premenopausal ones.

Absence of estrogen action notoriously leads to obesity in the healthy population [42], however this study revealed a larger fat mass increase compared to the 1.7% annual increase observed in healthy women transitioning from pre- to post-menopause, as reported in a recent paper [43].

It is well known that adjuvant chemotherapy is an independent predictor of weight gain in EBC patients [5–7]. In the present study chemotherapy was associated to the increase in FBM in univariable but not in the multivariable analysis. The two independent clinical predictors of increase of FBM after AI-therapy were adjuvant chemotherapy and premenopausal status.

The increase in fat tissue notably affected both the gynoid and, more significantly, the android components, leading to a marked increase in the fat ratio between the android and gynoid components. Although estrogen deficiency is expected to cause an unfavorable distribution of body fat, the changes observed in women participating in this study are remarkable. The relevance of this observation lies in the fact that variations in fat distribution are well-documented to increase the risk of adverse cardiovascular events [17]. The most significant change was observed in VAT, which increased by 18.0–20.0%. Since the availability of VAT was limited to a small number of patients, further investigations are necessary to confirm these data.

Notably, also LBM reduction observed in this study was much greater than the one observed in the healthy subjects in the aforementioned study (−0.2% per year) [43].

The results of this study show that body composition changes were highly variable across different body districts. FBM increased from 3.2% in the head to 10.9% in the trunk, while it had a more uniform increase in the limbs. LBM also had a variable reduction from −1.3% in the right arm to −3.8% and −3.9% in the trunk and head respectively. Different changes in body composition between trunk and limbs were observed in young women who underwent oophorectomy [44] or in menopausal women [45], confirming the results of the present study. In patients with prostate cancer undergoing androgen deprivation therapy, there has also been a heterogeneity in the change of body composition in relation to various body districts [46].

Based on the current findings, it is possible to develop supervised physical exercise programs targeting specific body districts where changes are more relevant, which could have practical implications.

This study provides the first comprehensive analysis of changes in body composition, both holistically and across various body regions, attributable to AI-treatment in a significant cohort of patients with EBC.

The strengths of the study include its prospective design, the relatively high number of patients enrolled, the use of DXA as a method to analyze body composition, and the study conduction in a single-center which allowed for DXA assessment with a single instrument and by the same group of radiologists. The short patient follow-up, the lack of a control group of post-menopausal EBC patients non-submitted to AIs are the main weaknesses.

In conclusion, the main novel findings of this study show that postmenopausal women given adjuvant AIs treatment undergo a significant increase in FBM and a significant reduction in LBM with a wide variability according to the different body districts. EBC patients under AIs are therefore at risk of sarcopenic obesity, which must be effectively counteracted. For these reasons, new strategies are needed and a supervised physical exercise focused on single body parts prone to show detrimental variations may be considered in EBC patients treated with AIs.

## Supplementary Information

Below is the link to the electronic supplementary material.

Supplementary file1 (PDF 65 KB)

Supplementary file2 (PDF 104 KB)

Supplementary file3 (PDF 96 KB)

Supplementary file4 (PDF 96 KB)

## Data Availability

The datasets generated and/or analysed during the current study are available from the corresponding author on reasonable request.
