# Supplementary figures and images for: Changes in body composition in early breast cancer patients treated with aromatase inhibitors
Source: J Endocrinol Invest. 2024 Jun 10;47(12):3119–28. doi: 10.1007/s40618-024-02401-7 (PMC11549134; doi:10.1007/s40618-024-02401-7)

Figure S1. Trial consort diagram.

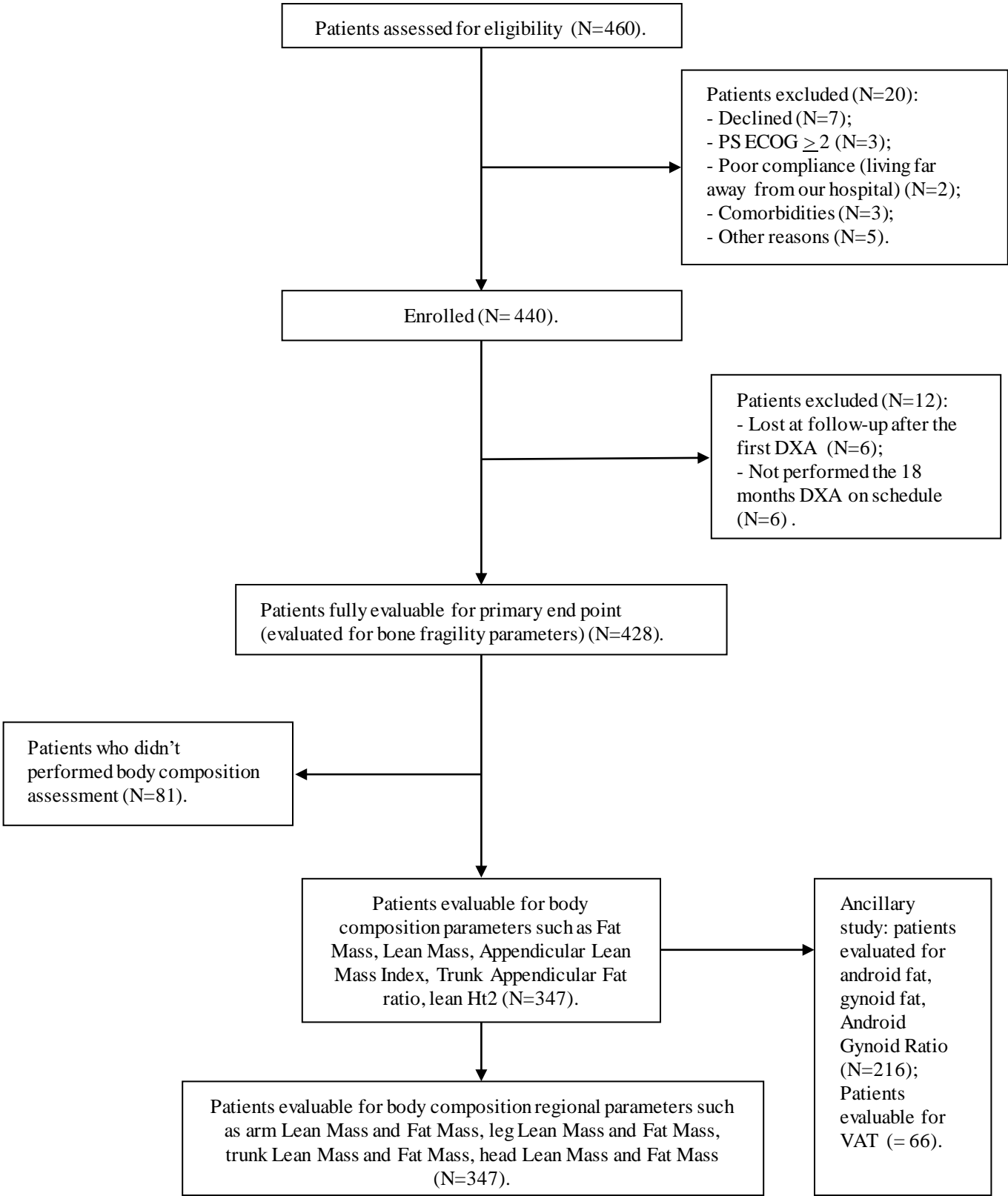

Supplement: Supplementary file 1 — Supplementary file1 (PDF 65 KB) [file 40618_2024_2401_MOESM1_ESM.pdf]
