# Supplementary material for: Changes in body composition in early breast cancer patients treated with aromatase inhibitors
Source: J Endocrinol Invest. 2024 Jun 10;47(12):3119–28. doi: 10.1007/s40618-024-02401-7 (PMC11549134; doi:10.1007/s40618-024-02401-7)
Supplement: Supplementary file 2 — Supplementary file2 (PDF 104 KB) [file 40618_2024_2401_MOESM2_ESM.pdf]

Journal of Endocrinological Investigation

Supplementary Information:

Changes in body composition in early breast cancer patients treated with aromatase inhibitors.

°Rebecca Pedersini[1,2], °Greta Schivardi[1], Lara Laini[1], Manuel Zamparini[1], Alessia Bonalumi[1], Pierluigi di Mauro[1], Sara Bosio[2], Vito Amoroso[1], Nicole Villa[1], Andrea Alberti[1], Nunzia Di Meo[3], Chiara Gonano[1], Barbara Zanini[4], Marta Laganà[1], Giuseppe Ippolito[1], Luca Rinaudo[5], Davide Farina[3], Maurizio Castellano[6], Carlo Cappelli[6], Edda Lucia Simoncini[2], *Deborah Cosentini[1], *Alfredo Berruti[1]

°These authors equally contributed and are co-primary authors

*These authors equally contributed and are co-senior authors

[1]Medical Oncology Department, ASST Spedali Civili of Brescia, Brescia, Italy

[2]SSVD Breast Unit, ASST Spedali Civili of Brescia, Brescia, Italy

[3]Department of Medical and Surgical Specialties, Radiological Sciences and Public Health, Medical Oncology, University of Brescia, ASST Spedali Civili, Brescia, Italy

[4]Department of Clinical and Experimental Sciences, University of Brescia, Italy

[5] Tecnologie Avanzate Srl, Turin, Italy

[6]Department of Internal Medicine and Endocrinology, University of Brescia, ASST Spedali Civili, Brescia, Italy

ESM_2. Characteristics of the enrolled patients

**Characteristics of the 428 patients**	**N° (%)**
Median age (range)	63 (28 – 84)
Physical activity<br>Yes<br>No	 99 (23.0%)<br>329 (77.0%)
Smoke<br>Yes<br>No	 102 (23.8%)<br>326 (77.2%)
Alcohol consumption<br>Yes<br>No	 86 (20.1%)<br>342 (80.9%)
pT<br>1<br>≥ 2	 285 (66.6%)<br>143 (44.4%)
pN<br>0<br>≥ 1	 258 (60.3 %)<br>170 (40.7%)
Histological type<br>No Special Type (NST)<br>Other	 294 (68.7%)<br>134 (42.3%)
Grading<br>G1 o G2<br>G3	 221 (51.6%)<br>207 (48.4%)
HER 2 status<br>Positive<br>Negative	 74 (17.3%)<br>354 (83.7%)
Chemotherapy<br>Yes<br>No	 185 (43.2%)<br>186 (56.8%)
**Characteristics of the 428 patients**	**Mean (95% CI)**
ER (%), mean (95% CI)	94.5 (93.2-95.9)
PgR (%), mean (95% CI)	59.3 (55.6-63.9)
Ki67 (%), mean (95% CI)	23.4 (21.9-24.8)

N°: number of patients; %: percentage of patients; CI: confidence interval; pT: pathological tumor stage; pN: pathological nodal status.
