# Supplementary material for: Changes in body composition in early breast cancer patients treated with aromatase inhibitors
Source: J Endocrinol Invest. 2024 Jun 10;47(12):3119–28. doi: 10.1007/s40618-024-02401-7 (PMC11549134; doi:10.1007/s40618-024-02401-7)
Supplement: Supplementary file 3 — Supplementary file3 (PDF 96 KB) [file 40618_2024_2401_MOESM3_ESM.pdf]

Journal of Endocrinological Investigation

Supplementary Information:

Changes in body composition in early breast cancer patients treated with aromatase inhibitors.

°Rebecca Pedersini[1,2], °Greta Schivardi[1], Lara Laini[1], Manuel Zamparini[1], Alessia Bonalumi[1], Pierluigi di Mauro[1], Sara Bosio[2], Vito Amoroso[1], Nicole Villa[1], Andrea Alberti[1], Nunzia Di Meo[3], Chiara Gonano[1], Barbara Zanini[4], Marta Laganà[1], Giuseppe Ippolito[1], Luca Rinaudo[5], Davide Farina[3], Maurizio Castellano[6], Carlo Cappelli[6], Edda Lucia Simoncini[2], *Deborah Cosentini[1], *Alfredo Berruti[1]

°These authors equally contributed and are co-primary authors

*These authors equally contributed and are co-senior authors

[1]Medical Oncology Department, ASST Spedali Civili of Brescia, Brescia, Italy

[2]SSVD Breast Unit, ASST Spedali Civili of Brescia, Brescia, Italy

[3]Department of Medical and Surgical Specialties, Radiological Sciences and Public Health, Medical Oncology, University of Brescia, ASST Spedali Civili, Brescia, Italy

[4]Department of Clinical and Experimental Sciences, University of Brescia, Italy

[5] Tecnologie Avanzate Srl, Turin, Italy

[6]Department of Internal Medicine and Endocrinology, University of Brescia, ASST Spedali Civili, Brescia, Italy

ESM_3. Risk factors of percentage change in FBM (g).

Characteristics of the 347 patients	Univariable analysis		Multivariable analysis	
	**B (95% CI)**	**P value**	**B (95% CI)**	**P value**
Age	-0.5 (-0.6; -0.3)	**<.001**	-0.2 (-0.4; -0.1)	**0.03**
Menopausal status				
Post-	1	**<.001**	1	**<.001**
Pre-	15.2 (10.8; 19.5)		10.4 (4.8; 16.0)	
Physical activity				
No	1	0.37		
Yes	-1.9 (-6.0; 2.3)			
Smoke				
No	1	0.25		
Yes	2.4 (-1.7; 6.4)			
Alcohol consumption				
No	1	0.34		
Yes	-2.0 (-6.2; 2.1)			
Chemotherapy				
No	1	**<.001**	1	0.44
Yes	5.3 (1.9; 8.8)		1.4 (-2.1; 4.9)	
Radiotherapy				
No	1	0.24		
Yes	-2.2 (-5.8; 1.5)			

FBM(g): fat body mass (grams); N°: number of patients; %: percentage of patients; CI: confidence interval; B: beta coefficient.
