# Supplementary material for: Changes in body composition in early breast cancer patients treated with aromatase inhibitors
Source: J Endocrinol Invest. 2024 Jun 10;47(12):3119–28. doi: 10.1007/s40618-024-02401-7 (PMC11549134; doi:10.1007/s40618-024-02401-7)
Supplement: Supplementary file 4 — Supplementary file4 (PDF 96 KB) [file 40618_2024_2401_MOESM4_ESM.pdf]

Journal of Endocrinological Investigation

Supplementary Information:

Changes in body composition in early breast cancer patients treated with aromatase inhibitors.

°Rebecca Pedersini[1,2], °Greta Schivardi[1], Lara Laini[1], Manuel Zamparini[1], Alessia Bonalumi[1], Pierluigi di Mauro[1], Sara Bosio[2], Vito Amoroso[1], Nicole Villa[1], Andrea Alberti[1], Nunzia Di Meo[3], Chiara Gonano[1], Barbara Zanini[4], Marta Laganà[1], Giuseppe Ippolito[1], Luca Rinaudo[5], Davide Farina[3], Maurizio Castellano[6], Carlo Cappelli[6], Edda Lucia Simoncini[2], *Deborah Cosentini[1], *Alfredo Berruti[1]

°These authors equally contributed and are co-primary authors

*These authors equally contributed and are co-senior authors

[1]Medical Oncology Department, ASST Spedali Civili of Brescia, Brescia, Italy

[2]SSVD Breast Unit, ASST Spedali Civili of Brescia, Brescia, Italy

[3]Department of Medical and Surgical Specialties, Radiological Sciences and Public Health, Medical Oncology, University of Brescia, ASST Spedali Civili, Brescia, Italy

[4]Department of Clinical and Experimental Sciences, University of Brescia, Italy

[5] Tecnologie Avanzate Srl, Turin, Italy

[6]Department of Internal Medicine and Endocrinology, University of Brescia, ASST Spedali Civili, Brescia, Italy

ESM_4. Risk factors of percentage change in LBM.

Characteristics of the 347 patients	Univariable analysis		Multivariable analysis	
	B (95% CI)	P value	B (95% CI)	P value
Age	0.0 (-0.0; 0.1)	0.61		
Menopausal status				
Post-	1	0.10	1	0.11
Pre-	-1.6 (-3.6; 0.4)		-1.6 (-3.6; 0.4)	
Physical activity				
No	1	0.09	1	0.09
Yes	1.6 (-0.2; 3.3)		1.5 (-0.2; 3.3)	
Smoke				
No	1	0.95		
Yes	-0.1 (-1.8; 1.7)			
Alcohol consumption				
No	1	0.71		
Yes	-0.3 (-2.1; 1.5)			
Chemotherapy				
No	1	0.97		
Yes	-0.0 (-1.5; 1.5)			
Radiotherapy				
No	1	0.65		
Yes	-0.4 (-1.9; 1.2)			

LBM(g): lean body mass; N°: number of patients; %: percentage of patients; CI: confidence interval; B: beta coefficient.
